# The Clinical Relevance of Unclassified Pulmonary Hypertension

**DOI:** 10.1016/j.chest.2025.12.058

**Published:** 2026-03-09

**Authors:** Teresa John, Lena Neuhuber, Philipp Douschan, Vasile Foris, Katarina Zeder, Gerhard Bachmaier, Horst Olschewski, Nikolaus Kneidinger, Gabor Kovacs

**Affiliations:** a Department of Internal Medicine, Division of Respiratory Medicine, Lung Research Cluster, ERN Lung Pulmonary Hypertension National Expert Center, Graz, Austria; b Institute for Medical Informatics, Statistics and Documentation, Medical University of Graz, Graz, Austria; c Channing Division of Network Medicine, Brigham and Women’s Hospital, Harvard Medical School, Boston, MA; d The University of Maryland—Institute of Health Computing, Bethesda, MD; e Sigmund Freud Private University, Faculty of Medicine, Charité University Medicine, Vienna, Austria; f Department of Pneumology, Intensive Care Medicine and Sleep Medicine, Charité University Medicine, Berlin, Germany; g University of Maryland School of Medicine, Division of Respiratory, Critical Care and Sleep Medicine, Baltimore, MD

To the Editor:

Pulmonary hypertension (PH) is a hemodynamic condition characterized by elevated mean pulmonary arterial pressure (mPAP) > 20 mm Hg, which may result from various underlying pathologies and is associated with poor prognosis.^1^^,^^2^ According to current guidelines, a subset of patients are categorized as having unclassified PH, defined as elevated mPAP but normal pulmonary vascular resistance (PVR) (≤ 2 Wood units) and pulmonary arterial wedge pressure (PAWP) ≤ 15 mm Hg,^1^^,^^2^ posing challenges in appropriate management. These individuals frequently exhibit increased pulmonary blood flow (PBF), necessitating a thorough evaluation for potential underlying conditions such as congenital heart disease, liver disease, airway disease, lung disease, or hyperthyroidism.^1^, ^2^, ^3^ The prevalence and clinical significance of unclassified PH is unknown and has not been systematically addressed.

## Methods

**Figure 1:**
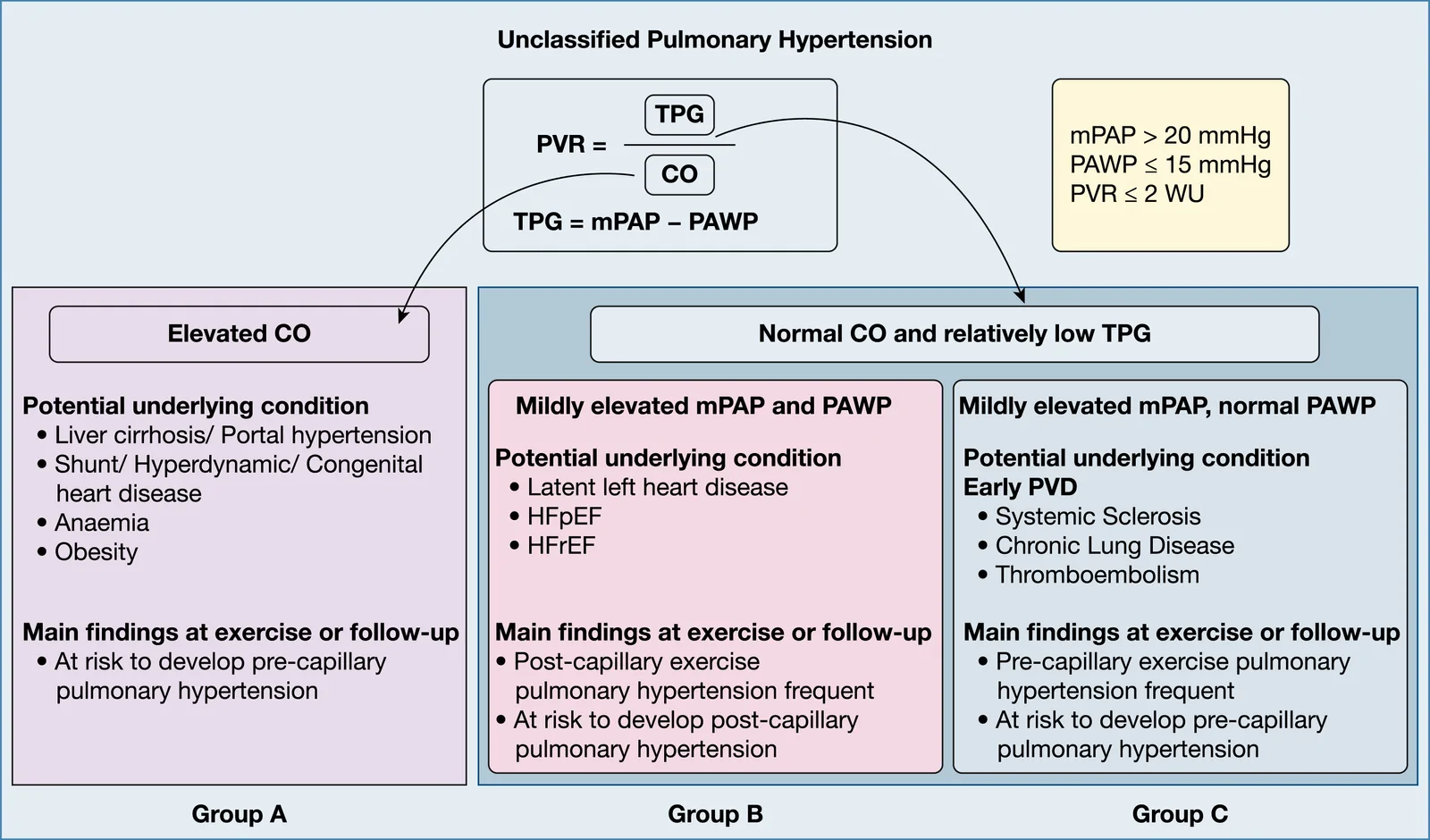
Illustration of underlying mechanisms leading to unclassified PH. CO = cardiac output; HFpEF = heart failure with preserved ejection fraction; HFrEF = heart failure with reduced ejection fraction; mPAP = mean pulmonary artery pressure, PAWP = pulmonary artery wedge pressure, PBF = pulmonary blood flow; PVD = pulmonary vascular disease; PVR = pulmonary vascular resistance; TPG = transpulmonary gradient. Created in BioRender. John, T. (2025) https://BioRender.com/3czpjwc

We conducted a retrospective analysis of patients from our PH clinic who underwent right heart catheterization (RHC) and clinical workup between 2005 and 2025, selecting those who met the hemodynamic criteria for unclassified PH. We focused on elucidating the underlying causes potentially leading to this hemodynamic condition and its clinical associations. We stratified patients based on the main hemodynamic feature explaining the presence of unclassified PH. In group A, we included participants with strongly elevated PBF (cardiac output [CO] > 8.0 L/min^1^), whereas patients with CO ≤ 8.0 L/min were divided based on their PAWP (group B, including patients with mildly elevated PAWP [13-15 mm Hg]),^4^, ^5^, ^6^ and group C with normal PAWP (<13 mm Hg) (Fig 1).

Values are presented as mean ± SD or as median and interquartile range for continuous data; categorical data are presented as absolute and relative numbers. For group comparisons, analysis of variance or Kruskall-Wallis tests were used, as appropriate. Significance level was defined as *P* < .05. Statistical analysis was performed by IBM SPSS Statistics 29. The study was approved by the local ethics committee of the Medical University of Graz (EK Nr 1079/2025).

## Results

Of 831 patients with PH in our database, n = 37 (4.5%) fulfilled the hemodynamic criteria of unclassified PH (Table 1). Ten patients (27%) had increased PBF (group A). Among these, 3 patients had significant left-to-right shunts, 3 had liver cirrhosis, 2 had anemia, 1 was on dialysis, and 1 had class III obesity (BMI > 40), representing their most likely explanations for elevated PBF.

**Table 1:** Patient Characteristics of the Cohort and the 3 Groups 1-3, Respectively

Variables	Total	Elevated PBF	Normal PBF		*P* Value
	Unclassified PH (n = 37)	Group A (n = 10)	Group B (mildly elevated PAWP) (n = 14)	Group C (normal PAWP) (n = 13)	
Male/female	21/16	7/3	5/9	4/9	.235
Age, y	61 ± 15	53 ± 18	62 ± 12	65 ± 13	.137
BMI	30.5 ± 6.6	30 ± 8	31 ± 7	31 ± 5	.828
Systolic BP, mm Hg	122 ± 16	114 ± 15	124 ± 15	127 ± 17	.134
Diastolic BP, mm Hg	64 ± 11	61 ± 10	66 ± 9	64 ± 13	.545
WHO functional class					.531
I	9 (24%)	3	3	3	
II	16 (44%)	3	5	8	
III	12 (32%)	4	6	2	
Laboratory testing					
Hemoglobin, g/dL	12.3 ± 2.1	10.6 ± 1.8	13.3 ± 2.0	12.6 ± 1.6	**.003**
NT-proBNP, pg/mL	250 (111-752)	243 (145-872)	476 (113-880)	219 (71-437)	.480
Bilirubin, mg/dL	0.5 (0.3-0.8)	0.7 (0.3-4.8)	0.5 (0.4-0.6)	0.5 (0.3-0.8)	.587
Albumin, g/dL	4.1 (3.5–4.3)	3.9 (3.3-4.3)	4.2 (3.9-4.3)	4.0 (3.5-4.5)	.254
GFR, mL/min/1.7	78 ± 30	88 ± 36	71 ± 30	78 ± 25	.444
Lung function testing					
FVC, % predicted	80 ± 17	82 ± 15	77 ± 16	83 ± 19	.579
FEV_1_, % predicted	78 ± 21	79 ± 16	74 ± 19	80 ± 26	.719
FEV_1_/FVC	76 (73-81)	76 (74-81)	77 (73-82)	77 (68-82)	.692
TLC, % predicted	94 ± 23	81 ± 32	92 ± 17	102 ± 21	.131
DlcocSB, % predicted	74 ± 17	69 ± 19	77 ± 16	75 ± 18	.573
DlcocVA, % predicted	89 ± 15	84 ± 15	95 ± 17	85 ± 10	.142
Echocardiographic parameters					
Resting TAPSE, mm	25 ± 6	27 ± 3	20 ± 6	29 ± 2	**.002**
Exercise testing					
6-min walk distance, m	368 ± 104	389 ± 107	350 ± 113	364 ± 93	.683
Right heart catheterization					
mPAP, mm Hg	22 (21-24)	24 (22-28)	23 (21-24)	21 (21-21)	**.002**
PAWP, mm Hg	12 (10-13)	11 (8-11)	14 (13-14)	10 (10-12)	**.001**
TPG, mm Hg	11 (9-12)	16 (12-17)	9 (8-10)	11 (10-12)	**.002**
RAP, mm Hg	7 (6-10)	7 (6-10)	8 (7-10)	6 (5-7)	.117
PVR, WU	1.8 (1.6-1.9)	1.6 (1.4-1.8)	1.7 (1.6-1.9)	1.8 (1.8-1.9)	**.022**
CO, L/min	6.7 ± 2.1	9.8 ± 1.7	5.5 ± 1.2	5.7 ± 0.4	**.001**
CI, L/min/m^2^	3.4 ± 1.2	4.8 ± 1.4	2.8 ± 0.5	3.0 ± 0.3	**.001**
Symptom-limited exercise right heart catheterization					
mPAP/CO slope, mm Hg/L/min	3.5 (2.5-5.1)	3.2 (2.7-3.2)	4.8 (3.4-11.4)	3.2 (2.3-4.2)	.179
PAWP/CO slope, mm Hg/L/min	2.0 (1.4-4.0)	2.0 (1.2-2.7)	3.5 (2.5-9.0)	1.5 (0.7-1.8)	**.004**
Comorbidities					
Cardiac	28	7	13	9	NA
CAD	5	1	4	0	NA
Arterial hypertension	21	4	9	9	NA
Shunt	3	3	0	0	NA
Atrial fibrillation	8	2	5	1	NA
Valve insufficiency > II	1	0	1	0	NA
Pulmonary	19	3	6	10	NA
COPD	7	2	2	3	NA
Asthma	2	0	1	1	NA
OSA	3	0	1	2	NA
Interstitial lung disease	9	0	2	7	NA
Chronic kidney disease	16	2	9	5	NA
GFR < 15 mL/m^2^/1.7 dialysis	1	1	0	0	NA
Liver	5	3	1	1	NA
Metabolic	19	5	7	7	NA
Diabetes	3	1	1	1	NA
Obesity (BMI > 30)	16	4	6	6	NA
Connective tissue disease	8	0	2	6	NA
Thromboembolism	6	1	3	2	NA

Normally distributed values are presented as mean ± SD; nonparametric variables are presented as median and interquartile range. Boldface indicates results that are statistically significant. CAD = coronary artery disease; CI = cardiac index; CO = cardiac output; DlcocSB = diffusing capacity of lung for carbon monoxide for single breath; DlcocVA = diffusing capacity of lung for carbon monoxide for alveolar volume corrected for hemoglobin; FEV_1_ = forced expiratory volume in 1 second the first record of expiration; GFR = glomerular filtration rate; mPAP = mean pulmonary artery pressure; NA = not applicable; NT-proBNP = N-terminal pro-B-type natriuretic peptide N-terminal pro-brain natriuretic peptide; PAWP = pulmonary artery wedge pressure; PBF = pulmonary blood flow; PH = pulmonary hypertension; PVR = pulmonary vascular resistance; RAP = right atrial pressure; sPAP = systolic pulmonary arterial pressure; TAPSE = tricuspid annular plane systolic excursion; TLC = total lung capacity; TPG = transpulmonary gradient; VO_2_ = oxygen uptake; WHO = World Health Organization; WU = Wood units.

The remaining 27 patients (73%) were characterized by a low transpulmonary gradient (Table 1). Of these, 14 patients (38%) had mildly elevated resting PAWP (13-15 mm Hg, group B), and n = 13 (35%) presented with normal PAWP (<13 mm Hg, group C). In group B, 13 of 14 patients had cardiovascular comorbidities, mostly systemic arterial hypertension, atrial fibrillation, and coronary artery disease (CAD).

In group C, mPAP was only mildly elevated (21 or 22 mm Hg). The most likely explanations for PH were systemic sclerosis (SSc) (n = 3), chronic pulmonary disease (n = 5), previous thromboembolic events (n = 1), metabolic syndrome (n = 1), chronic liver disease (n = 1), and CAD (n = 2).

In 29 of 37 patients with unclassified PH (5/10 of group A, 12/14 group B, and 12/13 group C), exercise RHC also was performed. Among these, 20 (69%) had exercise PH (mPAP/CO slope > 3 mm Hg/L/min) and 15 (52%) had a PAWP/CO slope > 2 mm Hg/L/min, suggesting latent left-sided heart disease.^7^ Of note, 12 of 14 patients with resting PAWP 13 to 15 mm Hg underwent exercise RHC, and 10 of them (83%) had a PAWP/CO slope > 2 mm Hg/L/min (Table 1). The assessment of exercise hemodynamics appeared to be clinically useful in patients with “unclassified” PH, because it contributed to the better understanding of the underlying pathophysiology. All patients later developing pre- or postcapillary PH presented with exercise PH, and in most patients with mildly elevated PAWP at rest the PAWP/CO slope was increased, confirming the role of latent left-sided heart disease.

A follow-up RHC was performed in 1 of 10 patients of group A. This patient continued to have unclassified PH with hyperdynamic circulatory state. In group B, follow-up RHC was performed in 3 of 14 participants: 1 developed isolated postcapillary PH, 1 precapillary PH, and 1 had no PH after optimization of heart failure therapy. In group C, 5 of 12 patients had follow-up RHC: 3 developed precapillary PH (all 3 had SSc), 1 combined pre- and postcapillary PH, and 1 had no PH anymore.

## Discussion

The term *unclassified PH* was introduced by the 2022 European Society of Cardiology and European Respiratory Society PH guidelines,^1^ but its prevalence and clinical significance have not been systematically addressed. In this study, we show that in a PH center, 4% to 5% of patients with PH may present with this hemodynamic condition and that they deserve clinical attention. By using a pragmatic approach, we identified specific groups of patients who may need regular follow-up and, potentially, specific management.

Increased PBF has been expected to be 1 of the main mechanisms leading to unclassified PH^1^^,^^2^ and was present in 27% of patients. The most frequent underlying conditions were chronic liver disease, left-to-right shunts, and anemia. From the clinical point of view, normal PVR in participants with unclassified PH and left-to-right shunts may suggest an indication for shunt closure.^8^

The largest group (38%) of patients with unclassified PH presented with mild PAWP elevation to 13 to 15 mm Hg and cardiovascular comorbidities—especially atrial fibrillation and CAD (Table 1). A postcapillary form of exercise PH characterized by a PAWP/CO slope >2 mm Hg/L/min was a frequent finding in this group, indicating an important role of latent left ventricular dysfunction contributing to unclassified PH.^4^

Patients with SSc (or other collagen vascular diseases) and unclassified PH deserve special attention, because they may present with early pulmonary vascular disease. In our cohort, we recognized 3 of 8 (38%) patients with SSc, who deteriorated hemodynamically during follow-up and developed more severe precapillary PH. The clinical relevance of unclassified PH in SSc also has been emphasized in recently published results of the Assessing the Spectrum of Pulmonary Hypertension Identified at a Referral Centre (ASPIRE) registry, showing that approximately 5% of patients with SSc and suspected PH had unclassified PH, out of whom 23% later developed precapillary PH, indicating disease progression.^9^

Remarkably, BMI was moderately elevated in all subgroups of unclassified PH, suggesting that obesity may contribute to mildly elevated mPAP in participants with normal PVR.^10^

Finally, among patients with normal PAWP and unclassified PH, chronic lung diseases were relatively frequent in our cohort. Although pulmonary disease is often accompanied by mild PH, our results indicate that PVR may remain in the normal range, whereas mPAP is already elevated.^3^

Our study has limitations. This is a single-center, retrospective analysis including a limited number of patients with unclassified PH. However, this may be mitigated by their thorough clinical and hemodynamic assessment using both resting and exercise RHC, allowing for a precise characterization of the underlying mechanisms of PH in this unique cohort.

In conclusion, this study highlights the heterogeneous nature of “unclassified PH,” characterized by distinct hemodynamic patterns, and provides a classification framework to enhance diagnosis and management. Exploration of the underlying clinical condition is essential in these patients, to provide optimal management and follow-up strategies.

## Financial/Nonfinancial Disclosures

The authors have reported to *CHEST* the following: T. J. reports personal fees and nonfinancial support from Janssen, nonfinancial support from Ferrer, nonfinancial support from MSD, nonfinancial support from Boehringer Ingelheim, personal fees from Roche, outside the submitted work; P. D. reports personal fees from Actelion, Janssen, Ferrer, GSK, Chiesi and nonfinancial support from Actelion, Astra Zeneca, Boehringer Ingelheim, GSK, MSD, Novartis, Menarini, Ferrer and Chiesi outside the submitted work; V. F. reports personal fees and nonfinancial support from Janssen, nonfinancial support from Boehringer Ingelheim, nonfinancial support from Astra Zeneca, personal fees and nonfinancial support from Chiesi, nonfinancial support from MSD, nonfinancial support from Ferrer, outside the submitted work; K. Z. reports a relationship with AstraZeneca Pharmaceuticals LP that includes consulting or advisory and travel reimbursement, a relationship with Grupo Ferrer Internacional SA that includes consulting or advisory and travel reimbursement. K. Z. reports a relationship with AOP Orphan Pharmaceuticals GmbH that includes travel reimbursement. H. O. reports grants and personal fees from MSD, personal fees from Janssen, personal fees from Ferrer, personal fees from Mondial, personal fees from MedUpdate, grants and personal fees from Iqvia, nonfinancial support from Ludwig Boltzmann Institute for Lung Vascular Research, outside the submitted work; G. K. reports grants, personal fees, and nonfinancial support from Janssen, grants, personal fees, and nonfinancial support from Boehringer-Ingelheim, personal fees from Bayer, personal fees and nonfinancial support from MSD, personal fees from Ferrer, personal fees from GSK, personal fees from Astra Zeneca, personal fees and nonfinancial support from AOP, personal fees from Chiesi, outside the submitted work. None declared (L. N., G. B., N. K.).
